# On asphericity of convex bodies in linear normed spaces

**DOI:** 10.1186/s13660-018-1624-z

**Published:** 2018-02-05

**Authors:** Nashat Faried, Ahmed Morsy, Aya M. Hussein

**Affiliations:** 1 Department of Mathematics, Faculty of Science, Ain Shams University, Cairo, Egypt; 2 Department of Mathematics, Faculty of Arts and Sciences at Wadi Addawasir, Prince Sattam Bin Abdulaziz University, Al-Kharj, Kingdom of Saudi Arabia

**Keywords:** 52A23, 52A10, 46B03, 57M35, 52A20, 32Q99, Dvoretzky theorem, Cross product of convex bodies, Asphericity, Convex set

## Abstract

In 1960, Dvoretzky proved that in any infinite dimensional Banach space *X* and for any $\epsilon> 0$ there exists a subspace *L* of *X* of arbitrary large dimension *ϵ*-iometric to Euclidean space. A main tool in proving this deep result was some results concerning asphericity of convex bodies. In this work, we introduce a simple technique and rigorous formulas to facilitate calculating the asphericity for each set that has a nonempty boundary set with respect to the flat space generated by it. We also give a formula to determine the center and the radius of the smallest ball containing a nonempty nonsingleton set *K* in a linear normed space, and the center and the radius of the largest ball contained in it provided that *K* has a nonempty boundary set with respect to the flat space generated by it. As an application we give lower and upper estimations for the asphericity of infinite and finite cross products of these sets in certain spaces, respectively.

## Introduction and basic definitions

In his profound and famous result, Dvoretzky 1960 [1, 2] proved that in any infinite dimensional Banach space *X* and for any $\epsilon> 0$ and natural number *n* there exists a subspace *L* of *X* with $\dim L=n$ such that $1 \leq d(L,\mathbb{R}^{n}) < 1+ \epsilon$, where *d* is the Banch–Mazur distance. The Banach–Mazur distance of two isomorphic Banach spaces *E* and *F*, is defined by $d(E,F)=\inf{ \Vert T \Vert \Vert T^{-1} \Vert }$, where the infimum is taken over all isomorphisms *T* from *E* onto *F* [3]. This result gave an affirmative answer to the conjecture raised by Grothendieck [4]: ‘Pour *n* and *ϵ* donnés, tout espace de Banach *E* de dimension assez grande contient un sous-espace isomorphe à *ϵ* près à l’espace de Hilbert de dimension *n*’. To get this result he proved that if *C* is a convex body (compact convex set with non-void interior) symmetric about the origin in an Euclidean space of sufficiently high dimension, there exists a *k*-dimensional subspace whose intersection with *C* has arbitrary small asphericity. This motivated us to give rigorous formulas which facilitate calculating the center and the radius of the smallest ball containing a set *K* in a linear normed space, and the center and the radius of the largest ball contained in it provided that *K* has a nonempty boundary set with respect to the flat space generated by it. Also, we use a formula to calculate the asphericity for each set has a nonempty boundary set with respect to the flat space generated by it. This allowed us to get lower and upper estimations for the asphericity of infinite and finite cross product of these sets in certain spaces, respectively.

### Definition 1.1

In linear normed space:

(i) A ball of largest diameter that lies entirely in a compact convex set *S* is called an inball of *S*. The ball of smallest diameter that enloses *S* is called the circumball [5].
(ii) A convex set *C* is called spherical to within *ϵ*, where $1 >\epsilon>0$, if there exist in the flat space generated by *C* (the smallest affine subspace that includes *C* as a subset) two concentric balls $B_{1}$ and $B_{2}$ of radii $r(1-\epsilon )$ and *r* such that $B_{1}\subseteq C \subseteq B_{2}$. The greatest lower bound of that *ϵ* having the above property is called the asphericity of *C* and is denoted by $\alpha(C)$ [1, 2].

### Definition 1.2

([6])

The set *A* in the Euclidean space $\mathbb{R}^{n}$ is called flat (linear variety, variety, and affine subspace) if whenever it contains two points, it also contains the entire line through them *i.e.* *A* is flat if $\lambda a+\mu b \in A$ whenever $a,b \in A$ and $\lambda+\mu =1$.

The condition for a set to be convex is less restrictive than for it to be flat, and so every flat set is a convex set. The term subspace of a linear space is used only for a flat set containing the origin.

To calculate the asphericity of any nonempty nonsingleton set *C* in a linear normed space such that $\operatorname {rbd}(C)$ is a nonempty set where $\operatorname {rbd}(C)$ is the boundary of *C* taken with respect to the flat space generated by the set *C*, we use the following formula:

1 $$\begin{aligned} \Gamma(C)= \inf_{x \in \operatorname {rint}(C)} \biggl[ \frac{ \sup_{y \in C} d(x,y)}{ \inf_{z \in \operatorname {rbd}(C)} d(x,z)} \biggr] . \end{aligned}$$

### Remark 1.3

([7])

Any nonempty nonsingleton bounded convex set *C* in finite dimensional linear normed space has nonempty $\operatorname {rbd}(C)$ set.

As an application, we give lower and upper estimations for the asphericity of infinite and finite cross product of sets in certain spaces, respectively, where each set has a nonempty boundary set with respect to the flat space generated by it.

## A technical lemma

In this section we mention a lemma that will be frequently used during the rest of the work.

### Lemma 2.1

(i) *Let* $(\alpha_{j}^{n})_{j=1}^{\infty}$, $n=1,2,3,\ldots$ , *be a countable family of summable sequences of real numbers*, *then for any* ${p \geq1}$ *we get*
  $$ \Biggl({\sum^{\infty}_{j=1} \inf _{n} \bigl\vert \alpha^{n}_{j} \bigr\vert } \Biggr)^{1/p} \leq\inf_{n} \Biggl({\sum ^{ \infty}_{j=1} \bigl\vert \alpha^{n}_{j} \bigr\vert } \Biggr)^{1/p} \leq \sup_{n} \Biggl({ \sum^{\infty}_{j=1} \bigl\vert \alpha^{n}_{j} \bigr\vert } \Biggr)^{1/p} \leq \Biggl({\sum^{\infty}_{j=1} \sup _{n} \bigl\vert \alpha^{n}_{j} \bigr\vert } \Biggr)^{1/p} $$
  *provided that* $\sum^{\infty}_{j=1} \sup_{n} \vert \alpha ^{n}_{j} \vert $ *exists*.
(ii) *Let* $(\alpha_{i})_{i \in I}$ *and* $(\beta_{j})_{j \in J}$ *be two bounded sequences of two independent sets of indices* *I* *and* *J*, *then for any* $p \geq1$ *we get*
  $$\begin{aligned}& \sup_{i \in I} \sup_{j \in J} \bigl({ \vert \alpha_{i} \vert + \vert \beta_{j} \vert } \bigr)^{1/p} = \Bigl({ \sup_{i} \vert \alpha_{i} \vert + \sup_{j} \vert \beta_{j} \vert } \Bigr)^{1/p} \quad \textit{and} \\& \inf_{i \in I} \inf_{j \in J} \bigl({ \vert \alpha_{i} \vert + \vert \beta_{j} \vert } \bigr)^{1/p} = \Bigl({ \inf_{i} \vert \alpha_{i} \vert + \inf_{j} \vert \beta_{j} \vert } \Bigr)^{1/p}, \end{aligned}$$
  *where the supremum and infimum in the LHSs are taken over all possible choices of* $i\in I$ *and* ${j\in J}$.
(iii) *For an infinite sequence of independent sets of indices* $J(n)$ *and for a countable number of bounded sequences* $(\alpha_{j _{n}}^{n})_{j_{n} \in J(n)}$, $n=1,2,3,\ldots$ , *we get for any* $p \geq1$
  $$\begin{aligned}& \sup_{j_{1} \in J(1)} \sup_{j_{2} \in J(2)}\cdots \Biggl({\sum ^{\infty }_{n=1} \bigl\vert \alpha^{n}_{j _{n}} \bigr\vert } \Biggr)^{1/p} = \Biggl(\sum^{\infty}_{n=1} \sup_{j_{n} \in J(n)} \bigl\vert \alpha^{n}_{j_{n}} \bigr\vert \Biggr)^{1/p} \quad \textit{and} \\& \inf_{j_{1} \in J(1)} \inf_{j_{2} \in J(2)} \cdots \Biggl({\sum ^{\infty }_{n=1} \bigl\vert \alpha^{n}_{j _{n}} \bigr\vert } \Biggr)^{1/p} = \Biggl({\sum^{\infty}_{n=1} \inf_{j_{n} \in J(n)} \bigl\vert \alpha^{n}_{j_{n}} \bigr\vert } \Biggr)^{1/p} \end{aligned}$$
  *provided that* $\sum^{\infty}_{n=1} \sup_{j_{n} \in J(n)} \vert \alpha^{n}_{j_{n}} \vert $ *exists*.

## Results and discussion

In the following we suggest two formulas to determine the radii and the centers of the smallest ball containing any nonempty nonsingleton set *C* in a linear normed space and the largest ball contained in it provided that $\operatorname {rbd}(C)$ is a nonempty set.

### Definition 3.1

Let *C* be a nonempty nonsingleton set in a linear normed space such that $\operatorname {rbd}(C) \neq \emptyset$ then

$$ \Delta(C) := \inf_{x\in C}\sup_{y\in C} \Vert x-y \Vert $$

is the radius of the smallest ball containing the set and

$$ \delta(C) := \sup_{x\in C}\inf_{y \in \operatorname {rbd}(C)} \Vert x-y \Vert $$

is the radius of the largest ball contained in the set.

### Proposition 3.2

*Let* *C* *be a nonempty nonsingleton compact set in a linear normed space such that* $\operatorname {rbd}(C) \neq \emptyset$ *then*

(i) *There exist* $x_{0}$ *and* $y_{0} \in C$ *satisfying* $\Delta(C) = \Vert x_{0}-y_{0} \Vert $.
(ii) *There exist* $x_{0}' \in C$ *and* $y_{0}' \in \operatorname {rbd}(C)$ *satisfying* $\delta(C) = \Vert x_{0}'-y_{0}' \Vert $.

### Proof

The proof is easy and will be omitted. □

### Remark 3.3

The center and the radius of the circumball is unique, but for the inball, the radius is unique but the center may not be unique. For example, the rectangle with vertices $(-2,-1)$, $(2,-1)$, $(-2,1)$ and $(2,1)$, any point belongs to the line segment on the *x*-axis between $(-1,0)$ and $(1,0)$ can be a center for an inball.

### Proposition 3.4

*The asphericity* $\alpha(C)$ *of a nonempty nonsingleton set* *C* *in a linear normed space such that* $\operatorname {rbd}(C)$ *is a nonempty set can be expressed as* $\alpha(C) = 1- \frac{1}{\Gamma(C)}$.

### Proof

From the definition of $\alpha(C)$, $\forall \eta>0$ $\exists \epsilon_{\eta}$, such that

$$ \alpha(C) \leq\epsilon_{\eta}< \alpha(C)+\eta $$

and $B_{1}\subset C \subset B_{2}$ where $B_{1}$ and $B_{2}$ are two concentric balls in the flat space generated by *C* with center $x_{0}$ (*i.e.* $x_{0}$ is an element of $\operatorname {rint}(C)$ (the interior of *C* with respect to the flat space generated by *C*)) and of radii $r(1-\epsilon_{\eta})$ and *r*. Then we can say that $B_{1}' \subset C \subset B_{2}$ where $B_{1}'$ and $B_{2}$ are two concentric balls in the flat space generated by *C* with center $x_{0}$ and of radii $r(1-\alpha(C)-\eta)$ and *r*. Then $r \geq\sup_{y \in C} d(x_{0},y)$ and $\inf_{z \in \operatorname {rbd}(C)} d(x_{0},z) \geq r(1-\alpha (C)-\eta)$. Then

$$ \Gamma(C) \leq\frac{ \sup_{y \in C} d(x_{0},y)}{ \inf_{z \in \operatorname {rbd}(C)} d(x_{0},z)} \leq\frac{r}{r(1-\alpha(C)-\eta)}. $$

So, $1-\alpha(C)-\eta\leq\frac{1}{\Gamma(C)}$. Therefore, $1-\frac{1}{\Gamma(C)} \leq\alpha(C)+\eta$.

On the other hand, $\forall \epsilon > 0$ $\exists x_{0}\in \operatorname {rint}(C)$, such that

$$ \Gamma(C) \leq\frac{ \sup_{y \in C}d(x_{0},y)}{ \inf_{z \in \operatorname {rbd}(C)} d(x_{0},z)} < \Gamma(C)+\epsilon. $$

Then $\sup_{y \in C}d(x_{0},y) < (\Gamma(C)+\epsilon) \inf_{z \in \operatorname {rbd}(C)} d(x_{0},z)$. Taking $r= \sup_{y \in C}d(x_{0},y)$ and $r(1-\epsilon) = \inf_{z \in \operatorname {rbd}(C)} d(x_{0},z)$, then $\frac {r}{\Gamma(C)+\epsilon}< r(1- \epsilon)$. So, $\frac{1}{\Gamma(C)+\epsilon} < 1-\epsilon$. So, $\epsilon< 1-\frac{1}{\Gamma(C)+\epsilon}$. Therefore, $\alpha(C)< 1-\frac{1}{\Gamma(C)+\epsilon}$. Since *ϵ* is arbitrary, $\alpha(C) \leq1-\frac{1}{\Gamma(C)}$. □

### Lemma 3.5

*For any set* *C* *in a linear normed space such that* $\operatorname {rbd}(C) \neq \emptyset$, *let* $C^{c}$ *be the complement of* *C* *taken with respect to the flat space generated by C*. *Then*

$$ \sup_{x\in C} \inf_{y\in \operatorname {rbd}(C) } d(x,y)= \sup _{x\in C} \inf_{y\in C^{c}} d(x,y). $$

### Proof

For every $x \in C$, let $\alpha_{x} = \inf_{y \in \operatorname {rbd}(C)} d(x,y)$, $\beta_{x} = \inf_{y \in C^{c}} d(x,y)$. Then for every $\epsilon>0 $ there exist $y_{\epsilon} \in \operatorname {rbd}(C)$ and $z_{\epsilon} \in C^{c}$ such that

$$\begin{aligned}& \alpha_{x} \leq d(x,y_{\epsilon}) < \alpha_{x} + \epsilon/2, \\& d(y_{\epsilon},z_{\epsilon}) < \epsilon/2. \end{aligned}$$

Then we have $\beta_{x} \leq d(x,z_{\epsilon}) \leq d(x,y_{\epsilon }) +d(y_{\epsilon},z_{\epsilon}) < \alpha_{x} + \epsilon$. On the other hand, for every $y \in C^{c}$ there exists $\epsilon_{y}$ such that

$$ d(y,x)-\epsilon_{y} \geq\alpha_{x}. $$

Therefore $\inf_{y \in C^{c}} d(y,x)\geq\alpha_{x}+ \inf_{y \in C^{c}} \epsilon_{y}$. Since $\inf_{y \in C^{c}} \epsilon_{y} = 0 $, we get the result. □

### Remark 3.6

For any nonempty nonsingleton set *C* in a linear normed space such that $\operatorname {rbd}(C) \neq\emptyset$ and $\operatorname {rint}(C) \neq\emptyset$

$$\begin{aligned} \Gamma(C) =& \inf_{ x \in \operatorname {rint}(C)} \biggl[ \frac{ \sup_{ y \in C} d(x,y)}{ \inf_{ z \in \operatorname {rbd}(C)} d(x,z)} \biggr] \\ =& \inf_{x \in int(C)} \biggl( \sup_{y \in C} d(x,y)* \sup_{z \in \operatorname {rbd}(C)} \frac{1}{d(x,z)} \biggr) \\ \geq& \inf_{x \in int(C)}\sup_{y \in C} d(x,y) * \inf _{x \in int(C)} \sup_{z \in \operatorname {rbd}(C)} \frac{1}{d(x,z)} \\ =& \frac{ \inf_{x \in int(C)} \sup_{y \in C} d(x,y)}{ \sup_{x \in int(C)} \inf_{z \in \operatorname {rbd}(C)} d(x,z)} \\ \geq& \frac{ \inf_{x \in C} \sup_{y \in C} d(x,y)}{ \sup_{x \in C} \inf_{z \in \operatorname {rbd}(C)} d(x,z)} =\frac{\Delta(C)}{\delta (C)}. \end{aligned}$$

### Definition 3.7

We say that a nonempty nonsingleton set *C* in a linear normed space such that $\operatorname {rbd}(C)$ and $\operatorname {rint}(C)$ are nonempty sets is regular if the center of its circumball is one of the centers for its inballs and in this case

$$ \frac{\Delta(C)}{\delta(C)}=\Gamma(C). $$

### Definition 3.8

([5])

A compact set *C* in $\mathbb{R}^{n}$ is said to be of constant width *δ* if every pair of parallel supporting hyperplanes are separated by a distance *δ*.

### Example 3.9

If $A \subset\mathbb{R}^{n}$ is a compact convex set of constant width *σ*, then *A* has a unique inball, which is concentric with its circumball, and $\sigma=R+r$ where *r* and *R* are the radii of the inball and the circumball, respectively. So *A* is a regular set.

### Theorem 3.10

*Let* $X_{1}, X_{2},\ldots$ *be a sequence of linear normed spaces*. *Let* $C_{i}$ *be a nonempty nonsingleton set in a linear normed space* $X_{i}$ *such that* $\operatorname {rbd}(C_{i}) \neq\emptyset$, $i= 1,2,3,\ldots$ , *and* $\operatorname {rint}(C_{i}) \neq\emptyset$ *for some i*. *Let* $\prod_{i=1} ^{\infty}C_{i} \subseteq l^{p}(X_{i})$ *where* $l^{p}(X_{i})$ *is the linear subspace of the Cartesian product* $X_{1} \times X_{2} \times X _{3}\cdots$ *equipped with the norm* $\Vert x \Vert _{p}=\sqrt [p]{\sum_{i=1}^{ \infty} \Vert x_{i} \Vert ^{p}}$ *and* $(\Delta (C_{i}))_{i=1}^{\infty}$ *and* $(\delta(C_{i}))_{i=1}^{\infty}\in l^{p}$ *then*

$$\begin{aligned}& 1.\quad \Delta \Biggl(\prod_{i=1}^{\infty}C_{i} \Biggr)= \Biggl( { \sum_{i=1}^{\infty} \Delta^{p}(C_{i})} \Biggr)^{1/p}, \\& 2.\quad \delta \Biggl(\prod_{i=1}^{\infty}C_{i} \Biggr) \leq \Biggl({ \sum_{i=1}^{\infty} \delta^{p}(C_{i})} \Biggr)^{1/p}, \\& 3.\quad \alpha \Biggl(\prod_{i=1}^{\infty} C_{i} \Biggr) \geq1- \Biggl[\sum_{i=1}^{\infty} \delta^{p}(C_{i}) / \sum_{i=1} ^{\infty} \Delta^{p}(C_{i}) \Biggr]^{1/p}. \end{aligned}$$

### Proof

Let $\Delta(C_{i})= \inf_{x\in C_{i}} \sup_{y\in C_{i}} d(x,y)$ and $\delta(C_{i})= \sup_{x\in C_{i}} \inf_{z\in C_{i}^{c}} d(x,z)$, $i=1,2,\ldots$ , be the radius of the circumball contaning $C_{i}$ and the radius of the inball contained in $C_{i}$, respectively. From Lemma 2.1(iii) we get

$$\begin{aligned} \Delta \Biggl( \prod_{i=1}^{\infty}C_{i} \Biggr) =& \inf_{ x_{1} \in C1,x_{2} \in C2,\ldots} \sup_{ y_{1} \in C1,y_{2} \in C2,\ldots} \Biggl({ \sum_{i=1}^{\infty} \Vert x _{i}-y_{i} \Vert ^{p}} \Biggr)^{1/p} \\ =& \inf_{ x_{1} \in C1,x_{2} \in C2,\ldots} \Biggl(\sum_{i=1}^{\infty} \sup_{y_{i} \in C_{i}} \Vert x_{i}-y_{i} \Vert ^{p} \Biggr)^{1/p} \\ =& \Biggl(\sum_{i=1}^{\infty}\inf _{ x_{i} \in Ci} \sup_{ y_{i} \in Ci} \Vert x_{i}-y_{i} \Vert ^{p} \Biggr)^{1/p} = \Biggl(\sum _{i=1}^{\infty}\Delta^{p}(Ci) \Biggr)^{1/p}. \end{aligned}$$

On the other hand, since $\prod_{i=1}^{\infty} \operatorname {rbd}(C_{i}) \subset \operatorname {rbd}( \prod_{i=1}^{\infty}C_{i})$ we get

$$\begin{aligned} \delta \Biggl( \prod_{i=1}^{\infty}C_{i} \Biggr) =& \sup_{x_{1} \in C1,x_{2} \in C2,\ldots} \inf_{y \in \operatorname {rbd}(\prod _{i=1}^{\infty}C_{i})} \Biggl(\sum _{i=1}^{\infty} \Vert x_{i}-y_{i} \Vert ^{p} \Biggr)^{1/p} \\ \leq& \sup_{x_{1} \in C1,x_{2} \in C2,\ldots} \inf_{y \in\prod _{i=1}^{\infty} \operatorname {rbd}(C_{i})} \Biggl(\sum _{i=1}^{\infty} \Vert x_{i}-y_{i} \Vert ^{p} \Biggr)^{1/p} \\ =& \sup_{x_{1} \in C1,x_{2} \in C2,\ldots} \Biggl({ \sum_{i=1}^{\infty} \inf_{ y_{i} \in \operatorname {rbd}(C_{i})} \Vert x_{i}-y_{i} \Vert ^{p} } \Biggr)^{1/p} \\ =& \Biggl(\sum_{i=1}^{\infty}\sup _{ x_{i} \in Ci} \inf_{ y_{i} \in \operatorname {rbd}(Ci)} \Vert x_{i}-y_{i} \Vert ^{p} \Biggr)^{1/p} = \Biggl( \sum _{i=1}^{\infty}\delta^{p}(C_{i}) \Biggr)^{1/p}. \end{aligned}$$

Now, we get a lower estimation for the asphericity $\alpha( \prod_{i=1}^{\infty}C_{i})$ of an infinite cross product of these sets. We have

$$\begin{aligned} \Gamma \Biggl(\prod_{i=1}^{\infty} C_{i} \Biggr) \geq \biggl( \frac{ { \sum_{i=1}^{\infty}\Delta^{p}(C_{i})} }{ { \sum_{i=1}^{\infty}\delta ^{p}(C_{i})} } \biggr) ^{1/p}. \end{aligned}$$

Hence,

$$\begin{aligned}& \alpha \Biggl( \prod_{i=1}^{\infty} C_{i} \Biggr) \geq1- { \biggl( \frac{ \sum_{i=1}^{\infty}\delta^{p}(C_{i}) }{ \sum_{i=1}^{\infty}\Delta^{p}(C_{i}) } } \biggr)^{1/p}. \end{aligned}$$

 □

### Lemma 3.11

*Let* $[\xi_{i} : i \in I]$, $[\eta_{j} : j \in J]$ *be two bounded families of real numbers then* $\max( \inf_{i \in I} \xi_{i}, \inf_{j \in J} \eta _{j}) = \inf_{i \in I, j \in J} \max(\xi_{i}, \eta_{j})$.

### Proof

Let $\alpha = \inf_{i \in I, j \in J} \max(\xi_{i}, \eta_{j})$. Therefore by taking a sequence ($\epsilon_{n}$) convergent to zero we get $\alpha\leq \max( \xi_{n}, \eta_{n}) < \alpha+ \epsilon_{n}$. Therefore, there exists either a sequence $\xi_{n_{k}}$ such that $\alpha\leq\xi_{n_{k}} < \alpha+ \epsilon_{n_{k}}$ or a sequence $\eta_{n_{k}}$ such that $\alpha\leq\eta_{n_{k}} < \alpha+ \epsilon_{n_{k}}$. So, we get either $\inf_{i \in I} \xi_{i} = \alpha$ or $\inf_{j \in J} \eta_{j} = \alpha$. So, we get $\max( \inf_{i \in I} \xi_{i}, \inf_{j \in J} \eta _{j}) \geq\alpha$.

On the other hand, $\beta= \max( \inf_{i \in I} \xi_{i}, \inf_{j \in J} \eta_{j}) \leq\max(\xi_{i}, \eta_{j})$ for all $i \in I$ and $j \in J$. Then $\max( \inf_{i \in I} \xi_{i}, \inf_{j \in J} \eta_{j}) \leq\inf_{i \in I, j \in J} \max(\xi_{i},\eta_{j})$. □

Similarly, we can prove the following.

### Lemma 3.12

1. *Let* $(\xi_{i_{j}}^{j})_{i_{j} \in I_{j}}$, $j = 1,2,\ldots,n$ *be any finite number of bounded families of real numbers*, *then* $\max_{1 \leq j \leq n} \inf_{i_{j} \in I_{j}} \xi_{i_{j}}^{j} = \inf_{i_{j} \in I_{j}} \max_{1 \leq j \leq n} \xi_{i_{j}}^{j}$.
2. *Let* $(\xi_{i}^{j})_{i \in I}$, $j = 1,2,\ldots,n$, *be any finite number of bounded families of real numbers*, *then* $\max_{1 \leq j \leq n} \inf_{i \in I} \xi_{i}^{j} \geq\inf_{i \in I} \max_{1 \leq j \leq n} \xi_{i}^{j}$.

### Theorem 3.13

*Let* $X_{1},X_{2},\ldots$ *be a sequence of linear normed spaces and* $C_{i}$ *be a nonempty nonsingleton set in* $X_{i}$ *such that* $\operatorname {rbd}(C_{i})$ *is a nonempty set*, $i=1,2,3,\ldots$ . *Let* $\prod C_{i} \subseteq l^{\infty}(X_{i})$ *where* $l^{\infty}(X_{i})$ *is the linear subspace of the Cartesian product* $X_{1} \times X_{2} \times X _{3}\cdots$ *equipped with the norm* $\Vert x \Vert _{\infty }=\sup_{i \in N} \Vert x _{i} \Vert $, *then* (i)

$$ \inf_{i} \bigl(\Gamma(C_{i}) \bigr) \leq\Gamma \biggl( \prod_{i} C_{i} \biggr), $$

(ii) *for any finite number of closed convex sets* $C_{i}$ *in a finite dimensional linear normed space* $X_{i}$ *such that* $\operatorname {rbd}(C_{i})$ *is a nonempty set*, $i=1,2,\ldots,n$, *and in particular*, *for any finite number of convex bodies* $C_{i}$ *in a finite dimensional linear normed space* $X_{i}$, $i=1,\ldots,n$,

$$ \Gamma{ \Biggl(\prod_{i=1}^{n} C_{i} \Biggr)} \leq\max \bigl(\Gamma(C_{1}),\ldots,\Gamma (C_{n}), \Delta(C_{1})/\delta(C_{n}),\ldots, \Delta(C_{n})/ \delta(C_{1}) \bigr)). $$

### Proof of (i)

For each $\eta>0$ there exists $\tilde{x} \in \operatorname {rint}( \prod_{i=1}^{\infty}C_{i})$ such that

$$\begin{aligned} \frac{\sup_{y \in\prod _{i=1}^{\infty}C_{i}} \Vert \tilde{x}-y \Vert }{ \inf_{z \in \operatorname {rbd}(\prod _{i=1}^{\infty}C_{i})} \Vert \tilde{x}-z \Vert } < \Gamma \Biggl(\prod_{i=1}^{\infty}C_{i} \Biggr) + \eta. \end{aligned}$$

Since $\prod_{i=1}^{\infty} \operatorname {rbd}(C_{i}) \subset \operatorname {rbd}(\prod_{i=1}^{\infty}C _{i})$,

$$\begin{aligned} \sup_{y \in\prod C_{i}} \Vert \tilde{x}-y \Vert < & \Biggl(\Gamma \Biggl( \prod_{i=1}^{\infty}C _{i} \Biggr)+\eta \Biggr) \inf_{z \in \operatorname {rbd}(\prod _{i=1}^{\infty}C_{i})} \Vert \tilde{x}-z \Vert \\ \leq& \Biggl(\Gamma \Biggl(\prod_{i=1}^{\infty}C_{i} \Biggr)+ \eta \Biggr) \inf_{z \in\prod _{i=1}^{\infty} \operatorname {rbd}(C_{i})} \Vert \tilde{x}-z \Vert \\ \leq& \Biggl(\Gamma \Biggl(\prod_{i=1}^{\infty}C_{i} \Biggr)+ \eta \Biggr) \Vert \tilde{x}-z \Vert \quad \text{for all } z \text{ in } \prod_{i=1}^{\infty} \operatorname {rbd}(C _{i}). \end{aligned}$$

For all $z \in\prod_{i=1}^{\infty} \operatorname {rbd}(C_{i})$,

$$ \sup_{y \in\prod C_{i}} \Vert \tilde{x}-y \Vert < \Biggl(\Gamma \Biggl( \prod_{i=1}^{\infty}C _{i} \Biggr)+ \eta \Biggr) \sup_{i=1}^{\infty} \Vert \tilde{x}_{i}-z_{i} \Vert . $$

Hence there exists $i_{0}$:

$$\begin{aligned}& \sup_{i=1}^{\infty}\sup_{y_{i} \in C_{i}} \Vert \tilde {x_{i}}-y_{i} \Vert < \Bigl( \Gamma \Bigl(\prod C_{i} \Bigr) +\eta \Bigr) \Vert \tilde{x_{i_{0}}}-z_{i_{0}} \Vert . \end{aligned}$$

So, $\sup_{y_{i} \in C_{i_{0}}} \Vert \tilde{x_{i_{0}}}-y_{i} \Vert < (\Gamma (\prod C_{i}) +\eta) \Vert \tilde{x_{i_{0}}}-z_{i_{0}} \Vert $ and hence we get

$$\begin{aligned}& \sup_{y_{i} \in C_{i_{0}}} \Vert \tilde{x_{i_{0}}}-y_{i} \Vert < \Bigl(\Gamma \Bigl( \prod C_{i} \Bigr) +\eta \Bigr) \inf_{z \in \operatorname {rbd}(C_{i_{0}})} \Vert \tilde {x_{i_{0}}}-z \Vert . \end{aligned}$$

Therefore,

$$ \inf_{i} \Gamma(C_{i}) \leq\frac{\sup_{y_{i} \in C_{i_{0}}} \Vert \tilde{x_{i_{0}}}-y_{i} \Vert }{\inf_{z \in \operatorname {rbd}(C_{i_{0}})} \Vert \tilde{x_{i_{0}}}-z \Vert } < \Gamma \Bigl(\prod C_{i} \Bigr). $$

 □

### Proof of (ii)

It suffices to prove this item for any two closed convex sets $C_{i}$ in a finite dimensional linear normed space $X_{i}$ such that $\operatorname {rbd}(C_{i})$ is a nonempty set, $i=1,2$. We have

$$\begin{aligned} \Gamma \Biggl( {\prod_{i=1}^{2}} C_{i} \Biggr) =& \inf_{(x_{i}) \in \operatorname {rint}(\prod_{i=1}^{2} C_{i})} \frac{ \sup_{(y_{i}) \in\prod_{i=1}^{2} C_{i}} \Vert x-y \Vert }{ \inf_{(z_{i}) \in \operatorname {rbd}(\prod_{i=1}^{2} C_{i})} \Vert x-z \Vert } \\ =& \inf_{(x_{i}) \in \operatorname {rint}(\prod_{i=1}^{2} C_{i})} \biggl[ \sup_{(y_{i}) \in\prod _{i=1}^{2} C_{i}} \sup _{i=1}^{2} \Vert x_{i}-y_{i} \Vert * \sup_{(z_{i}) \in \operatorname {rbd}(\prod_{i=1}^{2} C_{i})} \biggl(\frac{1}{ \sup_{i=1}^{2} \Vert x_{i}-z_{i} \Vert } \biggr) \biggr]. \end{aligned}$$

Since $\operatorname {rbd}(\prod_{i=1}^{2} C_{i}) = (\operatorname {rbd}(C_{1}) \times\overline{C_{2}}) \cup(\overline{C_{1}} \times \operatorname {rbd}(C_{2}))$ we have

$$\begin{aligned}& \begin{aligned} \alpha &= \sup_{(z_{i}) \in \operatorname {rbd}(\prod _{i=1}^{2} C_{i})} \biggl(\frac{1}{ \sup_{i=1}^{2} \Vert x_{i}-z_{i} \Vert } \biggr) \\ &= \sup_{\substack{z_{1} \in \operatorname {rbd}(C_{1}), z_{2} \in\overline{C_{2}} \text{ or} \\ z_{1} \in\overline{C_{1}}, z_{2} \in \operatorname {rbd}(C_{2})}} \biggl(\frac{1}{ \sup_{i=1}^{2} \Vert x_{i}-z_{i} \Vert } \biggr) \\ & = \max \biggl( \sup_{z_{1} \in \operatorname {rbd}(C_{1}), z_{2} \in\overline{C_{2}}} \frac {1}{ \sup_{i=1}^{2} \Vert x_{i}-z_{i} \Vert }, \sup _{z_{1} \in \overline{C_{1}}, z_{2} \in \operatorname {rbd}(C_{2})} \frac{1}{ \sup_{i=1}^{2} \Vert x_{i}-z_{i} \Vert } \biggr). \end{aligned} \end{aligned}$$

First suppose that $\alpha= \sup_{z_{1} \in \operatorname {rbd}(C_{1}), z_{2} \in \overline{C_{2}}} \frac{1}{ \sup_{i=1}^{2} \Vert x_{i}-z_{i} \Vert }$. Then, for all $\eta>0$ $\exists \overline{z_{1}}, \overline{z_{2}} \in \operatorname {rbd}(C _{1}), \overline{C_{2}}$, respectively, such that

$$ \frac{1}{ \Vert x_{1}-\overline{z_{1}} \Vert } \geq\inf_{i=1}^{2} \frac{1}{ \Vert x_{i}-\overline{z_{i}} \Vert } > \alpha- \eta, $$

we have $\sup_{z_{1} \in \operatorname {rbd}(C_{1})} \frac{1}{ \Vert x_{1}-z_{1} \Vert } > \alpha- \eta$.

Similarly, if we suppose that

$$\begin{aligned}& \alpha= \sup_{z_{1} \in\overline {C_{1}}, z_{2} \in \operatorname {rbd}(C_{2})} \frac{1}{ \sup_{i=1}^{2} \Vert x_{i}-z_{i} \Vert }\quad \text{then } \sup _{z_{2} \in \operatorname {rbd}(C_{2})} \frac{1}{ \Vert x_{2}-z_{2} \Vert } > \alpha- \eta. \end{aligned}$$

Then we have

$$\begin{aligned}& \max\biggl( \sup_{z_{1} \in \operatorname {rbd}(C_{1})} \frac{1}{ \Vert x_{1}-z_{1} \Vert }, \sup _{z_{2} \in \operatorname {rbd}(C_{2})} \frac{1}{ \Vert x_{2}-z_{2} \Vert }\biggr) > \alpha- \eta. \end{aligned}$$

Since *η* is arbitrary, we have

$$\begin{aligned}& \alpha\leq\max\biggl( \sup_{z_{1} \in \operatorname {rbd}(C_{1})} \frac{1}{ \Vert x_{1}-z_{1} \Vert }, \sup _{z_{2} \in \operatorname {rbd}(C_{2})} \frac{1}{ \Vert x_{2}-z_{2} \Vert }\biggr). \end{aligned}$$

The following is implied by Lemma 3.11 and Lemma 3.12:

$$\begin{aligned} \Gamma \Biggl(\prod_{i=1}^{2} C_{i} \Biggr) \leq& \inf_{(x_{i}) \in \operatorname {rint}(\prod_{i=1}^{2} C_{i})} \biggl[\max \Bigl( \sup_{y_{1} \in C_{1}} \Vert x_{1}-y_{1} \Vert , \sup_{y_{2} \in C_{2}} \Vert x_{2}-y_{2} \Vert \Bigr) \\ &{}* \max \biggl( \sup_{z_{1} \in \operatorname {rbd}(C_{1})} \frac{1}{ \Vert x_{1}-z_{1} \Vert }, \sup _{z_{2} \in \operatorname {rbd}(C_{2})} \frac{1}{ \Vert x_{2}-z_{2} \Vert } \biggr) \biggr] \\ =& \inf_{(x_{i}) \in \operatorname {rint}(\prod_{i=1}^{2} C_{i})} \max \biggl( \sup_{y_{1} \in C_{1}} \Vert x_{1}-y_{1} \Vert * \sup_{z_{1} \in \operatorname {rbd}(C_{1})} \frac{1}{ \Vert x_{1}-z_{1} \Vert }, \\ & \sup_{y_{2} \in C_{2}} \Vert x_{2}-y_{2} \Vert * \sup_{z_{2} \in \operatorname {rbd}(C_{2})} \frac{1}{ \Vert x_{2}-z_{2} \Vert }, \sup _{y_{1} \in C_{1}} \Vert x_{1}-y_{1} \Vert * \sup _{z_{2} \in \operatorname {rbd}(C_{2})} \frac{1}{ \Vert x_{2}-z_{2} \Vert }, \\ & \sup_{y_{2} \in C_{2}} \Vert x_{2}-y_{2} \Vert * \sup_{z_{1} \in \operatorname {rbd}(C_{1})} \frac{1}{ \Vert x_{1}-z_{1} \Vert } \biggr) \\ =& \inf_{(x_{i}) \in \operatorname {rint}(\prod_{i=1}^{2} C_{i})} \max \biggl(\frac{ \sup_{y_{1} \in C_{1}} \Vert x_{1}-y_{1} \Vert }{ \inf_{z_{1} \in \operatorname {rbd}(C_{1})} \Vert x_{1}-z_{1} \Vert }, \frac{ \sup_{y_{2} \in C_{2}} \Vert x_{2}-y_{2} \Vert }{ \inf_{z_{2} \in \operatorname {rbd}(C_{2})} \Vert x_{2}-z_{2} \Vert }, \\ & \frac{ \sup_{y_{1} \in C_{1}} \Vert x_{1}-y_{1} \Vert }{ \inf_{z_{2} \in \operatorname {rbd}(C_{2})} \Vert x_{2}-z_{2} \Vert }, \frac{ \sup_{y_{2} \in C_{2}} \Vert x_{2}-y_{2} \Vert }{ \inf_{z_{1} \in \operatorname {rbd}(C_{1})} \Vert x_{1}-z_{1} \Vert } \biggr) \\ \leq& \max \biggl( \inf_{x_{1} \in \operatorname {rint}(C_{1})} \frac{ \sup_{y_{1} \in C_{1}} \Vert x_{1}-y_{1} \Vert }{ \inf_{z_{1} \in \operatorname {rbd}(C_{1})} \Vert x_{1}-z_{1} \Vert }, \inf _{x_{2} \in \operatorname {rint}(C_{2})} \frac{ \sup_{y_{2} \in C_{2}} \Vert x_{2}-y_{2} \Vert }{ \inf_{z_{2} \in \operatorname {rbd}(C_{2})} \Vert x_{2}-z_{2} \Vert }, \\ & \inf_{\substack{ x_{1} \in \operatorname {rint}(C_{1}), \\ x_{2} \in \operatorname {rint}(C_{2})}} \frac{ \sup_{y_{1} \in C_{1}} \Vert x_{1}-y_{1} \Vert }{ \inf_{z_{2} \in \operatorname {rbd}(C_{2})} \Vert x_{2}-z_{2} \Vert }, \inf_{ \substack{ x_{1} \in \operatorname {rint}(C_{1}), \\ x_{2} \in \operatorname {rint}(C_{2})}} \frac{ \sup_{y_{2} \in C_{2}} \Vert x_{2}-y_{2} \Vert }{ \inf_{z_{1} \in \operatorname {rbd}(C_{1})} \Vert x_{1}-z_{1} \Vert } \biggr) \\ =& \max \biggl( \Gamma(C_{1}), \Gamma(C_{2}), \frac{ \inf_{x_{1} \in \operatorname {rint}(C_{1})} \sup_{y_{1} \in C_{1}} \Vert x_{1}-y_{1} \Vert }{ \sup_{x_{2} \in \operatorname {rint}(C_{2})} \inf_{z_{2} \in \operatorname {rbd}(C_{2})} \Vert x_{2}-z_{2} \Vert }, \\ &\frac{ \inf_{x_{2} \in \operatorname {rint}(C_{2})} \sup_{y_{2} \in C_{2}} \Vert x_{2}-y_{2} \Vert }{ \sup_{x_{1} \in \operatorname {rint}(C_{1})} \inf_{z_{1} \in \operatorname {rbd}(C_{1})} \Vert x_{1}-z_{1} \Vert } \biggr) \\ =& \max \biggl(\Gamma(C_{1}), \Gamma(C_{2}), \frac{\Delta(C_{1})}{ \delta(C_{2})}, \frac{\Delta(C_{2})}{\delta(C_{1})} \biggr). \end{aligned}$$

 □

## Conclusion

This study introduces a simple technique and rigorous formulas to facilitate calculating the asphericity for each set having a nonempty boundary set with respect to the flat space generated by it. Furthermore, the study gives a formula to determine the center and the radius of the smallest ball containing a nonempty nonsingleton set *C* in a linear normed space, and the center and the radius of the largest ball contained in it, provided that *C* has a nonempty boundary set with respect to the flat space generated by it. As an application we give lower and upper estimations for the asphericity of infinite and finite cross product of sets in certain spaces, respectively, where each set has a nonempty boundary set with respect to the flat space generated by it.

## References

[1] Dvoredsky A.P. (1961). Some results on convex bodies and Banach spaces. Proceedings of the International Symposium on Linear Spaces.

[2] Dvoretzky A. (1959). A theorem on convex bodies and applications to Banach spaces. Proc. Natl. Acad. Sci..

[3] Tomczak-Jaegermann N. (1989). Banach–Mazur Distances and Finite-Dimensional Operator Ideals.

[4] Grothendieck A. (1956). Sur certaines classes de suites dans les espaces de Banach et le théorème de Dvoretzky–Rogers. Bol. Soc. Mat. São Paulo.

[5] Lay S.R. (2007). Convex Sets and Their Applications.

[6] Webster R. (1994). Convexity.

[7] Bazaraa M.S., Shetty C.M. (2012). Foundations of Optimization.

